# *Ex situ* machine preservation of donor livers for transplantation: HOPE for all?

**DOI:** 10.1093/bjs/znab293

**Published:** 2021-09-03

**Authors:** O B van Leeuwen, R J Porte

**Affiliations:** Department of Surgery, Section of Hepatobiliary Surgery and Liver Transplantation, University of Groningen, University Medical Centre Groningen, Groningen, the Netherlands; Department of Surgery, Section of Hepatobiliary Surgery and Liver Transplantation, University of Groningen, University Medical Centre Groningen, Groningen, the Netherlands

## Abstract

Hypothermic oxygenated machine perfusion (HOPE) reduces ischaemia–reperfusion injury of donor livers and thereby improves outcomes after transplantation. End-ischaemic normothermic machine perfusion (NMP) enables assessment of hepatobiliary viability and selection of livers that would otherwise have been declined for transplantation. We advocate the combined use of (dual) HOPE and NMP for livers that are considered high risk, but may still be transplanted safely after *ex situ* resuscitation and assessment of hepatobiliary viability. Combined dual HOPE–NMP has the potential to substantially decrease the high rates of deceased donor liver discard.

Liver transplantation has become a victim of its own success. Shortage of organs is limiting access to this life-saving treatment. Despite this, many organs are refused for transplantation based on suboptimal quality and risk of early graft failure. Most deceased donor livers that are currently declined for transplantation are functioning well in the donor, but this can change after transplantation due to ischaemia–reperfusion injury that occurs during the procurement, preservation, and transplant procedures. The current standard of donor organ preservation is static cold ischaemic preservation after flush-out with a preservation solution. Although this method is sufficient for organs of optimal quality, it does not provide adequate protection against ischaemia–reperfusion injury in suboptimal donor organs. In particular, livers from donation after circulatory death (DCD) donors are more prone to substantial ischaemia–reperfusion injury that will result in graft-related complications after transplantation, such as primary graft non-function and non-anastomotic stricture (NAS) of the donor bile ducts. Both types of complication are more frequent after transplantation of DCD organs compared with donation after brain death (DBD) livers^1^.

A promising tool to reduce the risk of ischaemia–reperfusion injury after liver transplantation is *ex situ* oxygenated machine perfusion. Machine perfusion can be performed at various temperatures, and both hypothermic (8–12°C) and normothermic (35–37°) types of machine perfusion have entered clinical practice. Hypothermic and normothermic machine perfusion are not competing techniques. In fact, they serve different goals and can even be applied sequentially with additional benefits, as discussed below. A comparison of the characteristics of hypothermic and normothermic machine perfusion is provided in *Table 1.*

**Table 1:** Comparison of characteristics of hypothermic and normothermic liver machine perfusion

	HOPE (end-ischaemic)	NMP (end-ischaemic)
**Temperature (°C)**	8–12	35–37
**Oxygenation (kPa)**	>106	10–13
**Metabolic activity (%)**	∼10	∼100
**Mitochondrial resuscitation**	+++	+
**Protection against ischaemia–reperfusion injury**	+++	+/−
**Viability assessment**		
Hepatocellular	Perfusate FMN	Lactate, perfusate pH, bile production
Cholangiocellular	n.a.	Bile pH, bicarbonate, glucose
**Reduction in NAS**	+++	−
**Result of device failure**	Cold ischaemia	Warm ischaemia

HOPE, hypothermic oxygenated machine perfusion; NMP, normothermic machine perfusion; FMN, flavomononucleotide; n.a., not assessable; NAS, non-anastomotic stricture of donor bile duct.'+' depicts a positive effect, '-' depicts no effect.

## Hypothermic machine perfusion

An essential component of hypothermic machine perfusion is hyperoxygenation of a donor liver at low temperatures^2^. In hypothermia, cellular metabolism and adenosine 5′-triphosphate (ATP) demand are low. Despite the low temperature, mitochondrial function can be restored when a high oxygen tension is provided, resulting in restoration of the mitochondrial electron chain and a significant reduction in the levels of accumulated succinate and reduced nicotinamide adenine dinucleotide^2^. As a consequence, the mitochondrial generation of ATP is restored. This resuscitation of mitochondria takes away the main driving force of ischaemia–reperfusion injury. Hypothermic oxygenated machine perfusion (HOPE) can be performed by either portal vein perfusion alone or by dual perfusion of both the portal vein and hepatic artery (DHOPE). End-ischaemic (D)HOPE is effective when applied for a few hours, shortly before implantation of the donor liver^3^^,^^4^. (D)HOPE not only protects the liver parenchyma against ischaemia–reperfusion injury, but also the bile ducts^5^. In a randomized controlled European multicentre trial^4^, DHOPE was shown to result in a significant reduction in postreperfusion syndrome, early allograft dysfunction, and NAS after DCD transplantation compared with conventional static cold storage.

## Normothermic machine perfusion

The term normothermic machine perfusion (NMP) is used for two preservation techniques: end-ischaemic normothermic machine *perfusion* (back to base) and normothermic machine *preservation* (NMP starting in donor hospital). During NMP, the donor liver is metabolically active, which allows assessment of hepatobiliary viability before transplantation. In the first RCT^6^ of normothermic machine *preservation*, NMP was not primarily used for graft assessment and selection, but rather to study the potential preservation benefits of NMP compared with static cold storage. Recipients of a NMP-preserved liver had lower peak serum aspartate aminotransferase levels and less early allograft dysfunction than recipients of a liver after static cold storage. This technique brings logistical challenges, as a donor surgeon needs to be able to make arterial reconstructions and connect the donor liver safely to the perfusion device. Furthermore, during transportation, device failure could lead to warm ischaemia of the liver and the risk of discard. These logistical difficulties have favoured the end-ischaemic or ‘back-to-base’ application of NMP^7^. However, end-ischaemic NMP is unavoidably associated with ischaemia–reperfusion injury, although this may be less severe than during warm reperfusion in the recipient. This may explain why end-ischaemic NMP of high-risk livers, such as those from DCD donors, results in high rates of NAS after transplantation^8^^,^^9^.

## Combined DHOPE and normothermic machine perfusion

As it has become evident that a short period of end-ischaemic (D)HOPE protects against ischaemia–reperfusion injury, whereas end-ischaemic NMP enables viability assessment, but does not provide similar protection against ischaemia–reperfusion injury, we have proposed the combination of these two machine perfusion modalities. If (D)HOPE and NMP are considered as complementary techniques, the sequential use of these two types of machine perfusion could maximize the utilization of high-risk donor livers^10^. So far, over 60 high-risk, nationwide discarded, donor livers have been perfused with sequential DHOPE and NMP at this hospital, with excellent graft survival and low rates of post-transplant cholangiopathy (3 per cent). Growing confidence in the combined DHOPE–NMP protocol resulted in a utilization rate of over 80 per cent for the past 20 consecutive livers.

## Groningen machine perfusion protocol

**Fig. 1:**
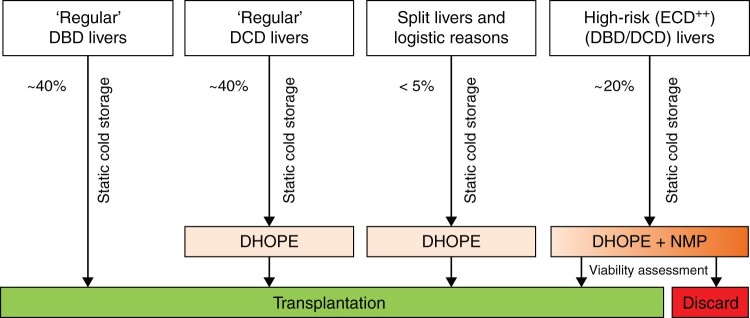
Schematic overview of liver machine perfusion protocols used at University Medical Centre Groningen DBD, donation after brain death; DCD, donation after circulatory death; ECD , extended-criteria donor; DHOPE, dual hypothermic oxygenated machine perfusion; NMP, normothermic machine perfusion.

As it has become clear that hypothermic and normothermic machine perfusion are not competing techniques, but tools that serve different goals, both techniques have been implemented in clinical practice in our hospital (*Fig. 1*). DHOPE is used for all DCD livers, as well as for DBD livers where the cold ischaemia time threatens to become too long (for example in combined lung–liver transplantation^11^). In addition, *ex situ* split procedures are now performed during DHOPE, to avoid extension of the cold ischaemia time^12^. For high-risk donor livers (both DBD and DCD) that are declined nationwide for regular transplantation, combined DHOPE–NMP is used. Moreover, in the setting of a clinical trial, the aim is to avoid night-time surgery and use DHOPE for extended organ preservation until surgery can be initiated during daytime (Netherlands Trial Register, NTR8740).

Machine perfusion provides transplant surgeons with an important tool to improve outcomes after liver transplantation, as well as to increase the number of suitable donor livers for transplantation. HOPE reduces ischaemia–reperfusion injury of donor livers and thereby improves outcomes after transplantation. In addition, HOPE facilitates transplant logistics by reducing the cold ischaemia time. End-ischaemic NMP enables assessment of hepatobiliary viability and selection of livers that would otherwise have been declined for transplantation. In contrast to HOPE, end-ischaemic NMP does not protect against ischaemia–reperfusion injury and related complications, such as post-transplant cholangiopathy. Therefore, we advocate the combined use of (D)HOPE and NMP for livers that are considered high risk, but may still be transplanted safely after *ex situ* resuscitation and hepatobiliary viability assessment. Combined DHOPE–NMP has the potential to substantially decrease the high rates of deceased donor liver discard .

*Disclosure*. The authors declare no conflict of interest.
